# Supplementary material for: Serum proteomics links suppression of tumor immunity to ancestry and lethal prostate cancer
Source: Nat Commun. 2022 Apr 1;13:1759. doi: 10.1038/s41467-022-29235-2 (PMC8975871; doi:10.1038/s41467-022-29235-2)
Supplement: Supplementary file 1 — Supplementary Information [file 41467_2022_29235_MOESM1_ESM.pdf]

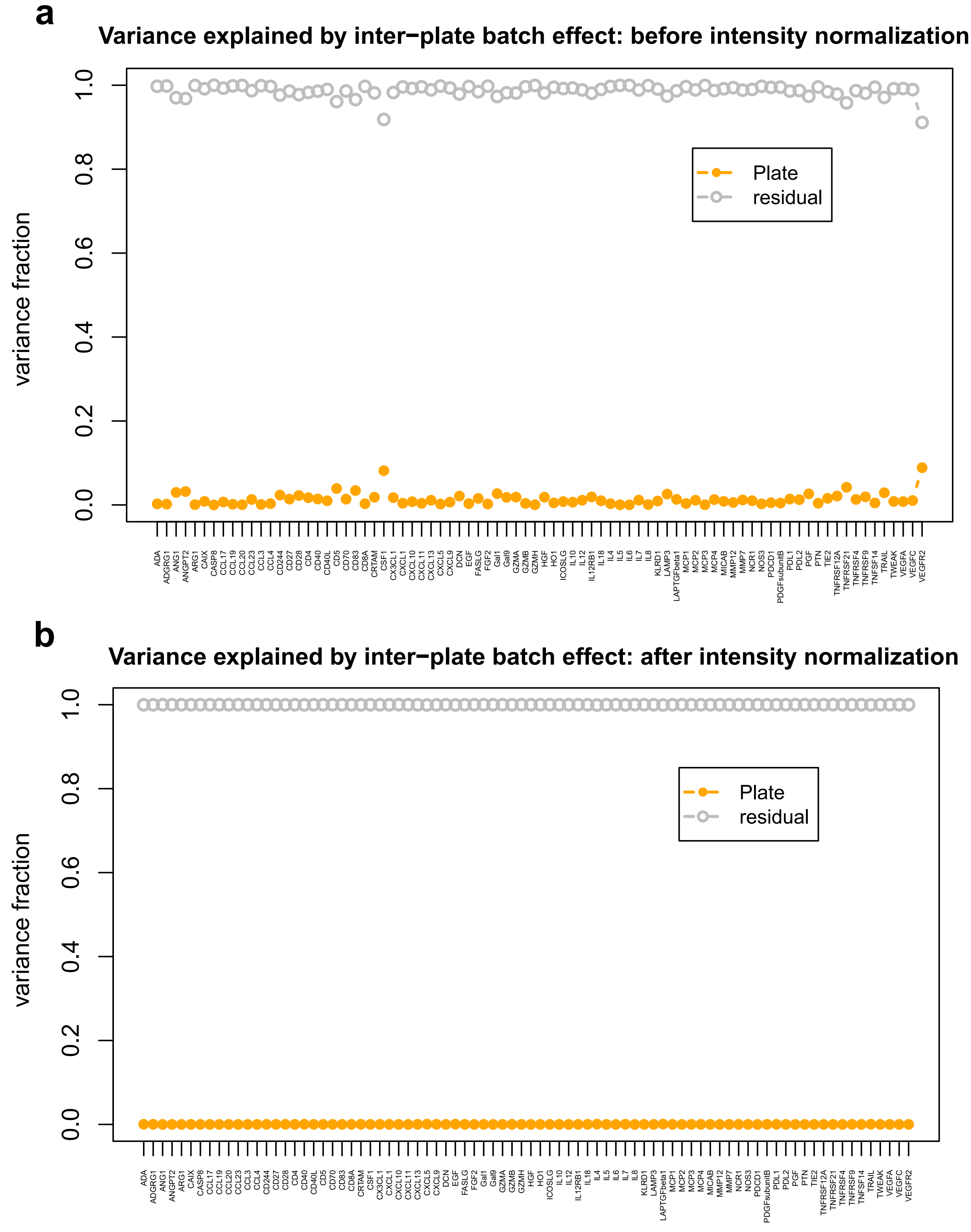

Supplementary Fig. 1. Variance explained by the inter-plate batch effect. The proportion of variance attributable to inter-plate batch variation and residual variation **(a)** before intensity normalization and **(b)** after intensity normalization is presented. Source data are provided as a Source Data file.

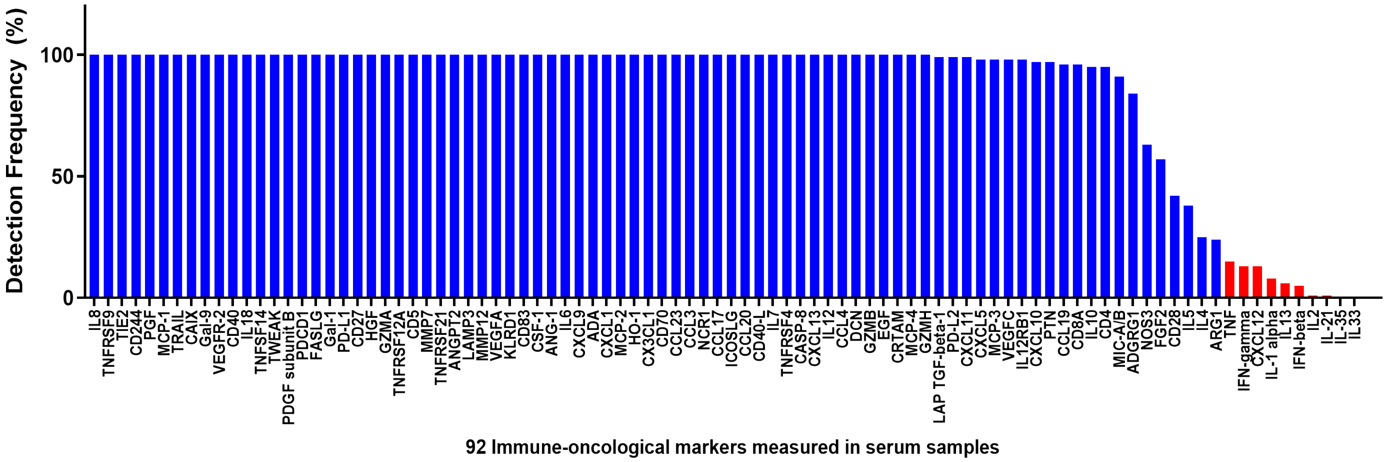

Supplementary Fig. 2. Detection frequencies of 92 immuno-oncological markers measured in 2937 serum samples. Out of the 92 proteins, 61 were detected in 100% of the samples tested whereas 78 were detected in >50% of the samples. IL33, IL35, IL21, IL2, IFNbeta, IL13, IL1alpha, CXCL12, IFNgamma, and TNF were detected in less than 20% of the samples (red bars), hence the remaining 82 proteins were used for subsequent analyses. Source data are provided as a Source Data file.

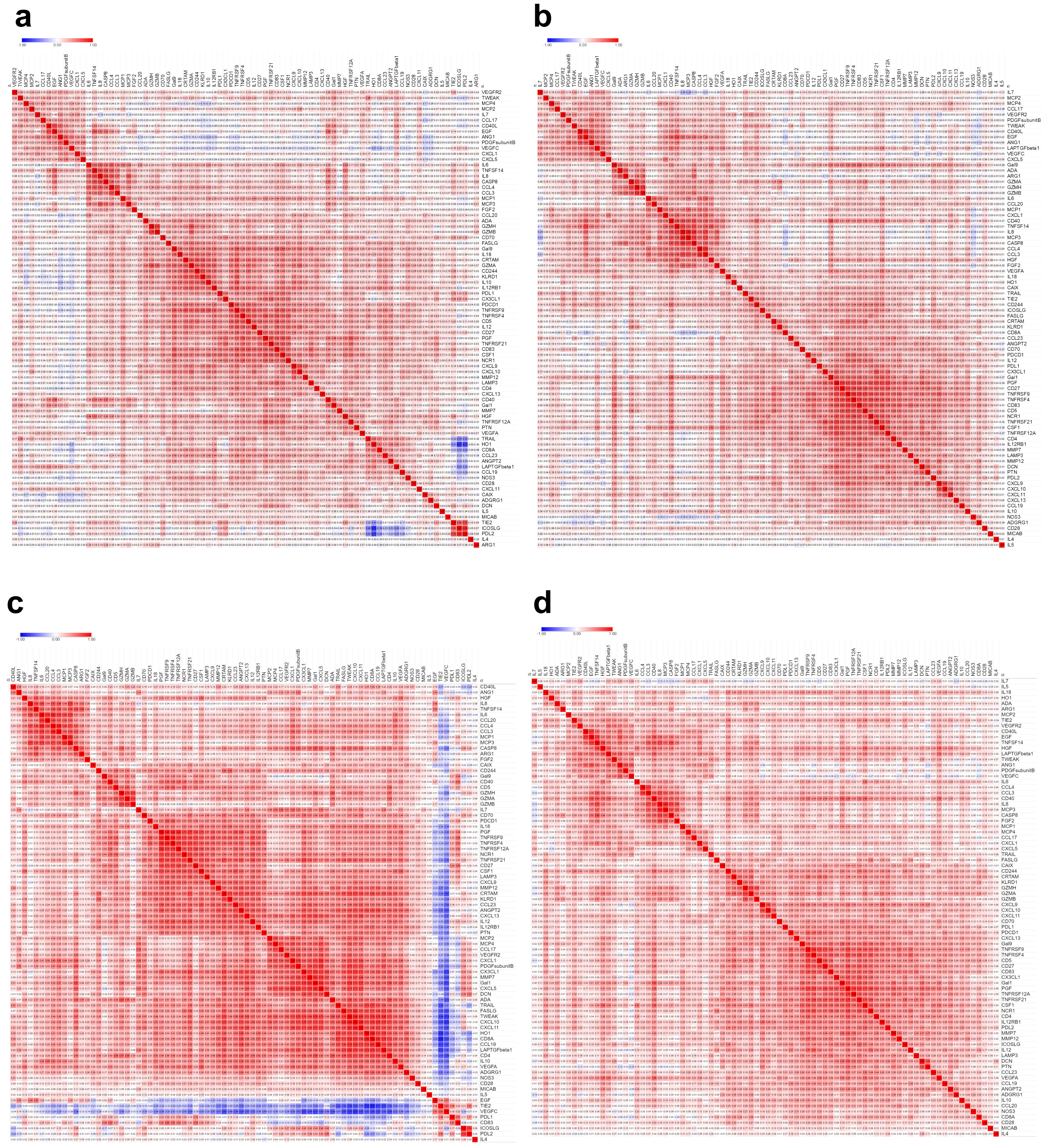

Supplementary Fig. 3. Correlation matrix presenting Pearson pairwise correlations for each of the 82 serum protein pairs. Pearson pairwise correlations were estimated for each serum protein pairs in **(a)** Ghanaian (n=654) and **(b)** EA (n=454) population controls and **(c)** Ghanaian (n=489) and **(d)** EA (n=425) prostate cancer cases. Source data are provided as a Source Data file.

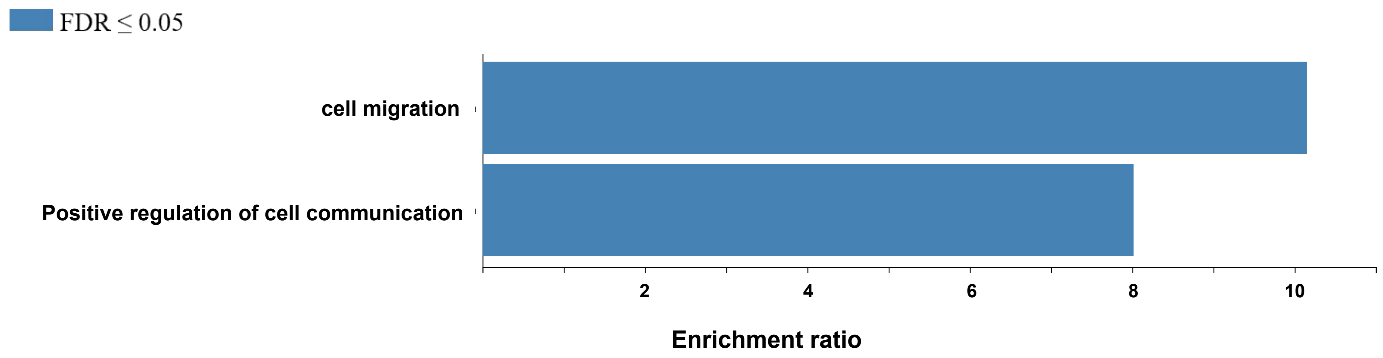

Supplementary Fig. 4. Gene ontology (GO) enrichment analysis. Over-Representation Analysis (ORA) was performed to identify the biological functions over-represented by the serum proteins that were positively associated with aging in all three population cohorts (Ghanaian, AA, and EA). Source data are provided as a Source Data file.

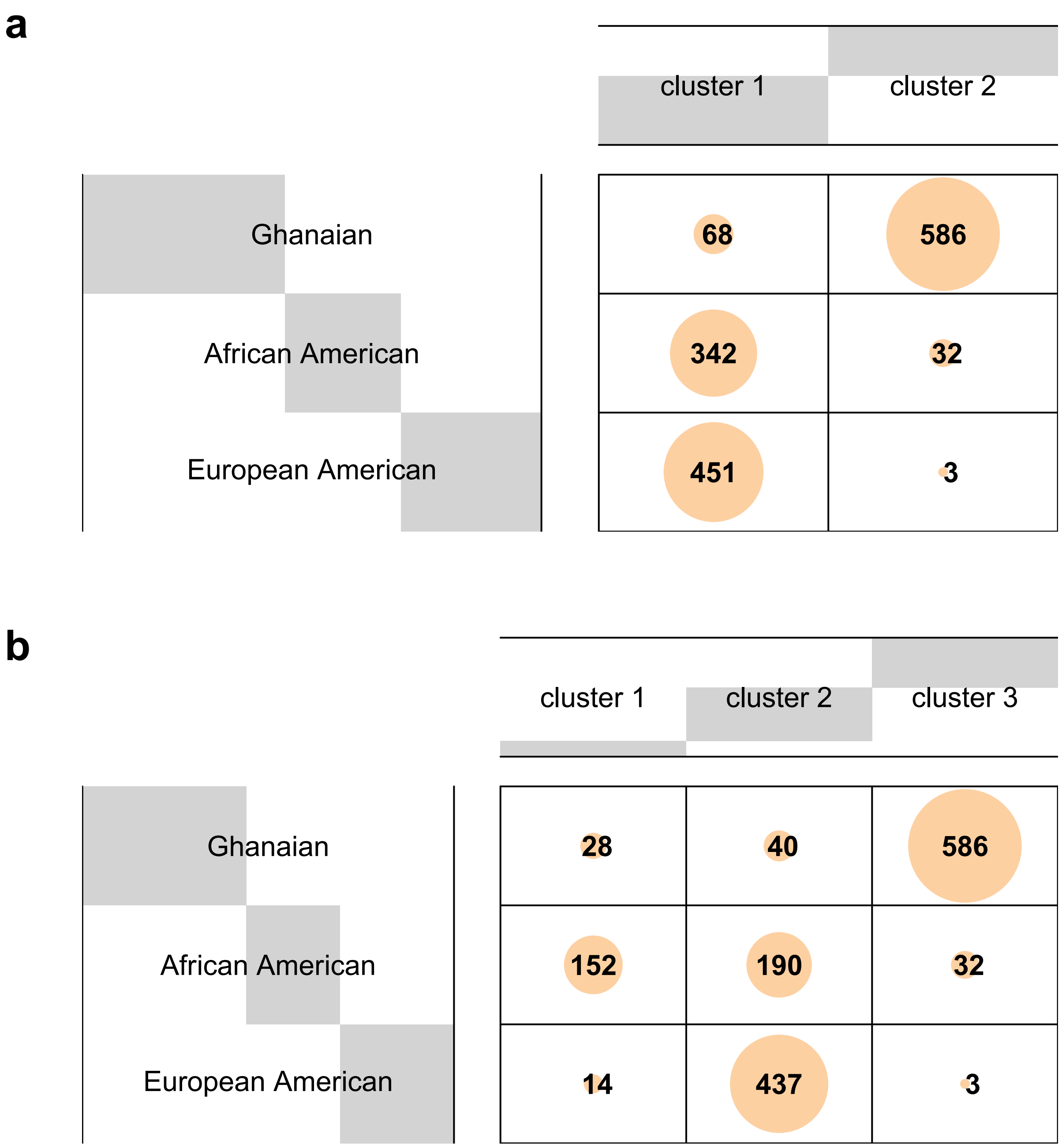

Supplementary Fig. 5. Clusters based on 82-protein profiles are enriched by population group (654 Ghanaian, 374 AA, and 454 EA). The dendrogram obtained from hierarchical clustering was cut to extract K clusters, yielding the contingency tables of cluster assignments vs. population groups shown for **(a)** K=2 and **(b)** K=3. For K=2, cluster 1 contains most EA and AA men, while cluster 2 contains most Ghanaian men. For K=3, EA are mostly grouped in cluster 2, AA are chiefly shared between clusters 1 and 2, while most Ghanaian men are in cluster 3. Cluster enrichment by population group was confirmed by Fisher's and chi-squared tests (two-sided p<1.e-10). Source data are provided as a Source Data file.

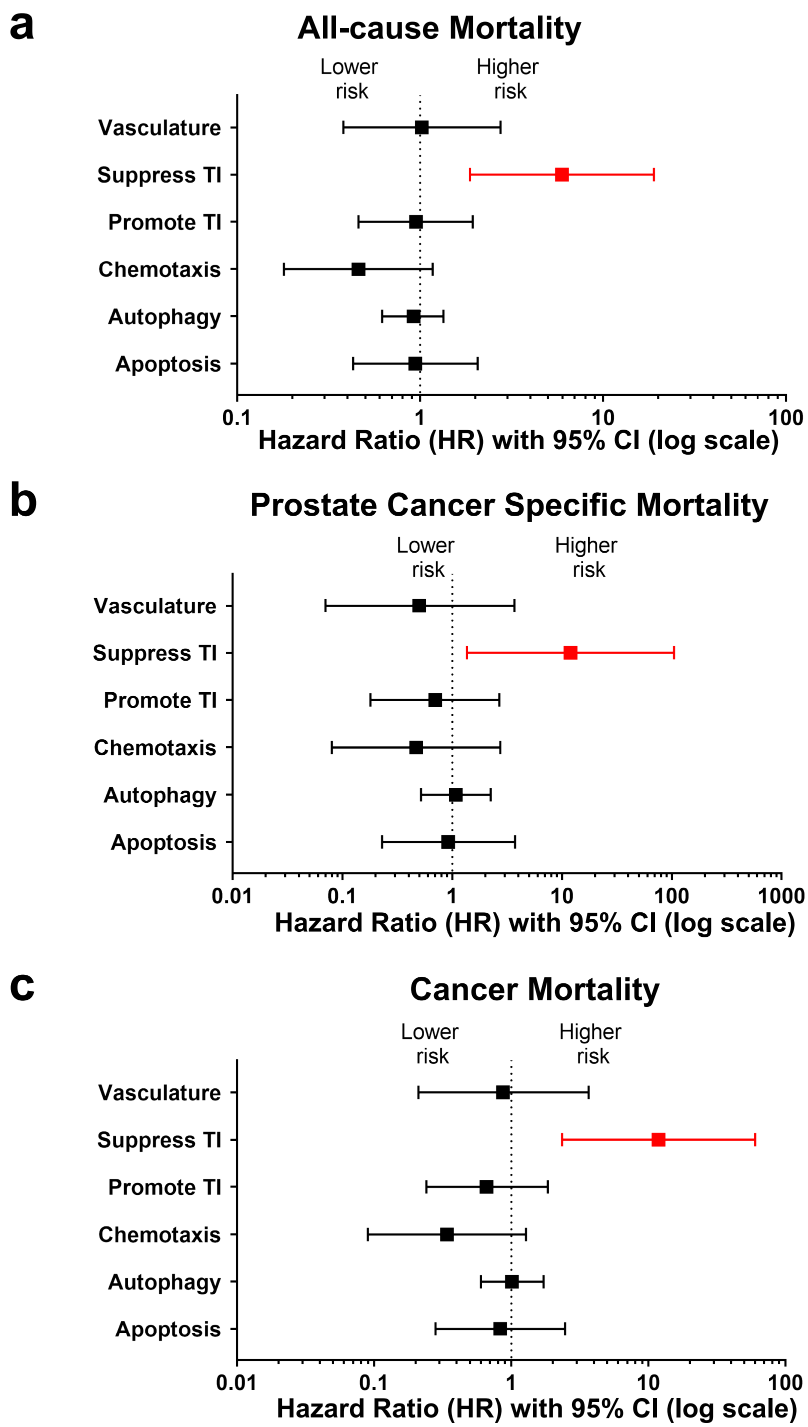

Supplementary Fig. 6. Suppression of the tumor immunity pathway associates with survival of prostate cancer patients. We assessed the association of the six pathways defined by the 82 immune-oncology markers with all-cause mortality (n=202), prostate cancer-specific mortality (n=57), or mortality due to any cancerafter a prostate cancer diagnosis (n=103) out of the 819 prostate cancer patients followed. The pathway scores were evaluated as continuous predictor variables. Suppression of tumor immunity pathway was distinctively associated with all-cause mortality **(a)**, prostatecancer-specific mortality **(b)**, or mortality due to any cancer after a prostate cancer diagnosis **(c)**. Multivariable Cox regression analyses were used to assess if the pathways were independently associated with survival of prostate cancer patients in the NCI-Maryland study. For **(a-c)**, the hazard ratios(HRs) were adjusted for age at study entry (years), body-mass index (BMI, kg/m2), self-reported race (AA/EA), education (high school or less, some college, college, professschool), income (less than $10k, $10-30K, $30-60K, $60-90k, greater than $90k), smoking history (never,ormer, current), diabetes (no/yes), aspirin use (no/yes), treatment (0=none, 1=surgery, 2=radiotherapy, 3=hormone, 4=combination), and NCCN risk score. The HRs indicatethe change in risk of dying when the biological process z-score value increases by 1 while holding all the other biological processes' z-scores and covariates constant. Data are presented as hazard ratios +/- 95% Confidence Intervals (CI). Source data are provided as a Source Data file. TI= tumor immunity.

a

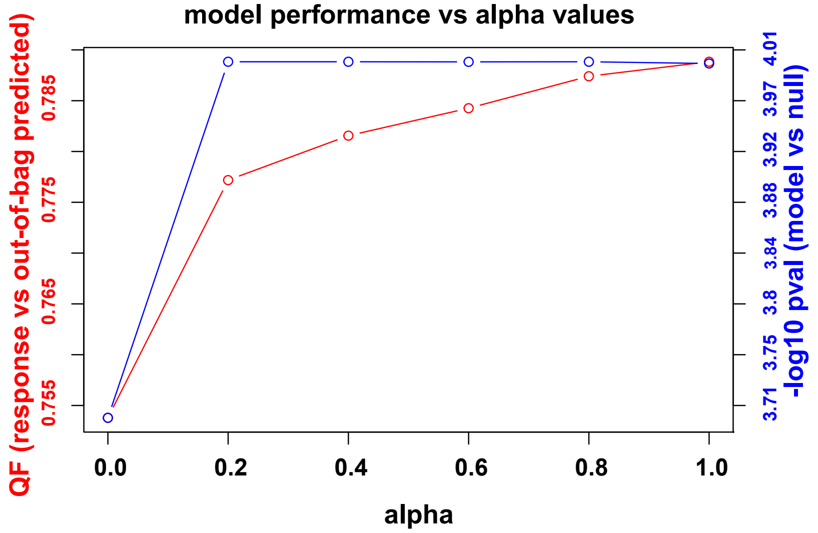

b

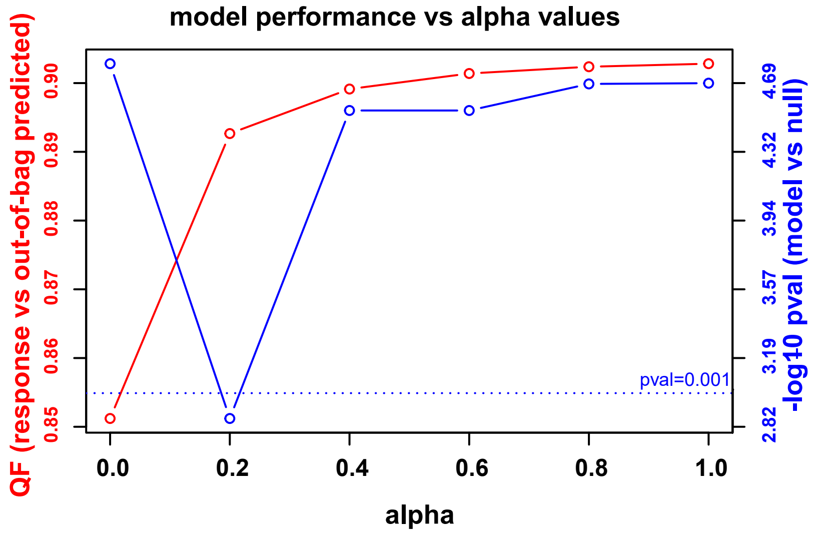

Supplementary Fig. 7. Performance of regularized Cox regression models across alpha. Cross-validated, regularized Cox regression models with different elastic net mixture parameter from ridge (alpha=0) to lasso (alpha=1) were implemented to identify a predictive proteomic signature of lethal prostate cancer for AA patients **(a)** without and **(b)** with NCCN risk score. Performance across alpha is shown with the 5-fold cross-validated quality function (QF) or concordance (red) and statistical significance (emperical one-sided p-value of model vs null) (blue). Lasso regression model (alpha=1) had the best out-of-bag performance. Source data are provided as a Source Data file.

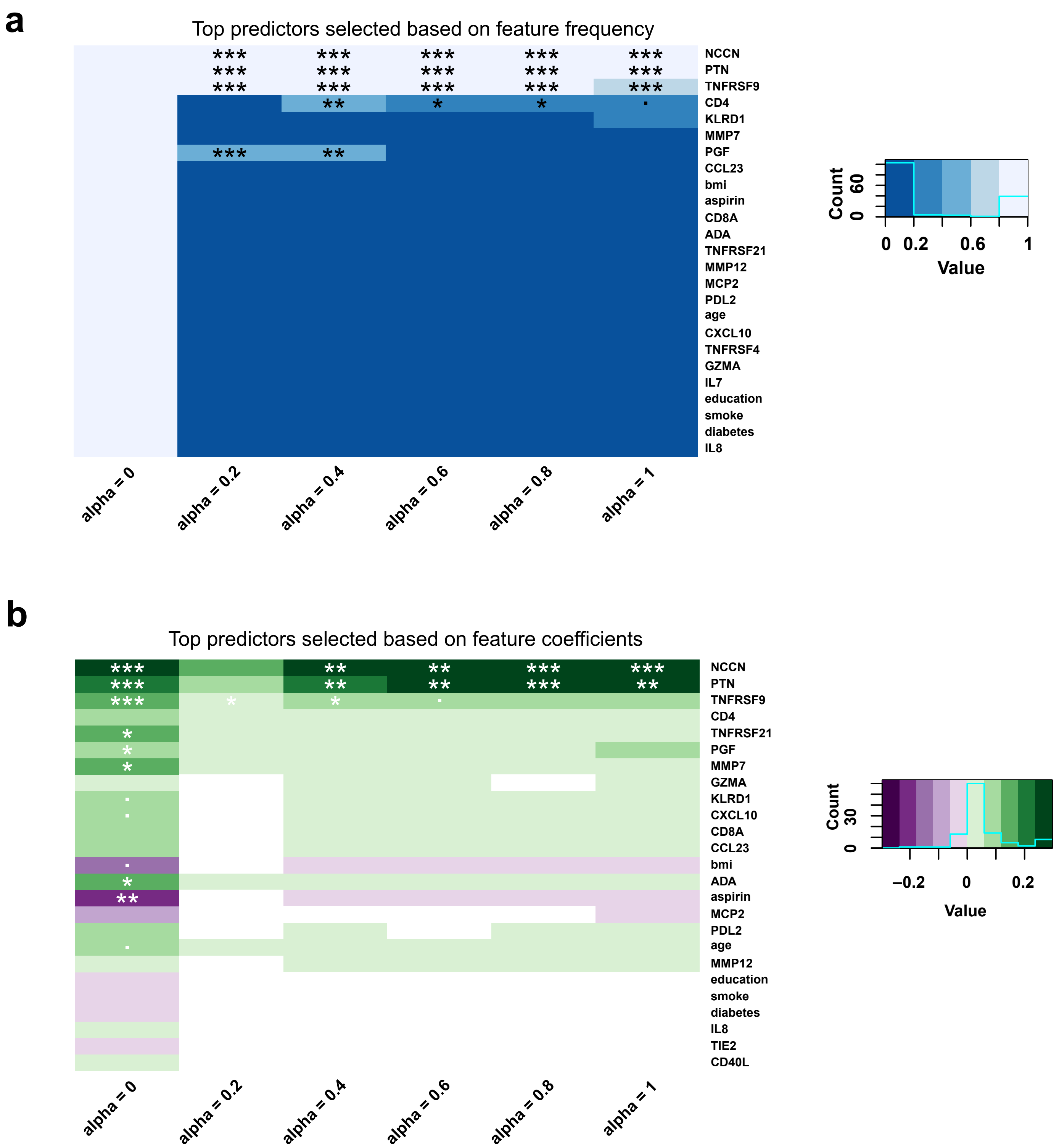

Supplementary Fig. 8. NCCN and analytes pleiotrophin (PTN) and TNFRSF9/CD137/4-1BB are the most predictive features for lethal prostate cancer in AA patients. Cross-validated, regularized Cox regression models with different elastic net mixture parameters from ridge (alpha=0) to lasso (alpha=1) were implemented to identify a predictive proteomic signature. One-sided *P* values were obtained empirically by comparing feature frequencies and coefficients against those obtained from random permutations of the response. **(a)** Heatmaps of feature frequencies across alpha. Features were ranked by *P* value for alpha=1. **(b)** Heatmaps of feature coefficients across alpha. Features were ranked by *P* value for alpha=1. *P* value significance was coded as <0.001 (***), <0.01 (**), <0.05 (*), and <0.1 (.). The exact *P* values are found in the Source Data file.Source data are provided as a Source Data file.

Supplementary Table 1. Characteristics of prostate cancer cases and population controls of the NCI-Maryland and NCI-Ghana Studies

Demographics	Controls Ghanaian (n=654)	Controls AA[a] (n=374)	Controls EA[b] (n=454)	Cases Ghanaian (n=489)	Cases AA (n=394)	Cases EA (n=425)
Age[c]						
Median (IQR[d]) in years	59 (11)	64 (10)	66.5 (13)	70 (11)	63 (11)	65 (11)
BMI						
Mean(SD[e]) in kg/m2	24.3 (4.4)	29.6 (5.4)	27.8 (4.7)	25.6 (5.1)	28.0 (5.2)	28.0 (4.3)
Education for NCI-MD, *N* (%)						
High school or less	NA	107 (29)	83 (18)	NA	183 (46)	110 (26)
Some college	NA	109 (29)	93 (20)	NA	130 (33)	110 (26)
College	NA	84 (22)	136 (30)	NA	53 (13)	103 (24)
Graduate	NA	73 (20)	142 (31)	NA	27 (7)	102 (24)
Did not provide	NA	1 (<1)	-	NA	1 (<1)	-
Education for NCI-Ghana, *N(%)*						
None	75 (11)	NA	NA	39 (8)	NA	NA
Primary	47 (7)	NA	NA	16 (3)	NA	NA
Middle / junior secondary	292 (45)	NA	NA	132 (27)	NA	NA
Secondary/ senior secondary	125 (19)	NA	NA	99 (20)	NA	NA
Higher	86 (13)	NA	NA	190 (39)	NA	NA
Did not provide	29 (4)	NA	NA	13 (3)	NA	NA
Annual household income, *N* (%)						
Less than $10,000	NA	18 (5)	5 (1)	NA	58 (15)	16 (4)
$10,000- $29,999	NA	63 (17)	37 (8)	NA	131 (33)	79 (19)
$30,000 - $59,999	NA	78 (21)	111 (24)	NA	94 (24)	89 (21)
$60,000-$90,000	NA	90 (24)	105 (23)	NA	41 (10)	86 (20)
Greater than $90,000	NA	86 (23)	168 (37)	NA	32 (8)	130 (31)
Don't know/Refused	NA	39 (10)	28 (6)	NA	38 (10)	25 (6)
**Baseline Health Factors**						
Smoking status[f], *N* (%)						
Never	346 (53)	149 (40)	191 (42)	321 (66)	112 (28)	171 (40)
Former	186 (28)	153 (41)	216 (48)	150 (31)	152 (39)	187 (44)
Current	94 (14)	70 (19)	42 (9)	10 (2)	127 (32)	65 (15)
Did not provide	28 (4)	2 (<1)	5 (1)	8 (2)	3 (<1)	2 (<1)
Diabetes, *N* (%)						
No	602 (92)	260 (70)	377 (83)	397 (81)	282 (72)	360 (85)
Yes	45 (7)	114 (30)	77 (17)	87 (18)	112 (28)	65 (15)
Did not provide	7 (1)	-	-	5 (1)	-	-
Regular aspirin use, *N* (%)						
No	570 (87)	179 (48)	173 (38)	347 (71)	227 (58)	193 (45)
Yes	66 (10)	195 (52)	281 (62)	78 (16)	167 (42)	232 (55)
Did not provide	18 (3)	-	-	64 (13)	-	-
Gleason score, *N* (%)						
Gleason score 2-6/ low grade	NA	NA	NA	149 (30)	159 (40)	187 (44)
Gleason score 7/ intermediate grade	NA	NA	NA	175 (36)	167 (42)	165 (39)
Gleason score 8-10/ high grade	NA	NA	NA	158 (32)	68 (17)	73 (17)
Unavailable	NA	NA	NA	7 (1)	-	-
Stage[g], *N* (%)						
I	NA	NA	NA	NA	62 (16)	97 (23)
II	NA	NA	NA	NA	280 (71)	264 (62)
III	NA	NA	NA	NA	23 (6)	41 (10)
IV	NA	NA	NA	NA	29 (7)	23 (5)
NCCN risk groups[h], *N* (%)						
Low	NA	NA	NA	NA	61 (15)	98 (23)
intermediate	NA	NA	NA	NA	217 (55)	211 (50)
High/ Very high	NA	NA	NA	NA	91 (23)	95 (22)
Regional/ Metastatic	NA	NA	NA	NA	25 (7)	21 (5)
PSA[i]						
Median (IQR) in ng/ml	0.98 (1.46)	0.4 (0.6)	0.4 (0.6)	51.9 (101)	6.8 (7.6)	6.0 (4.9)

[a] AA: African-American; [b] EA: European American; [c] Age at study interview; [d] IQR: Interquartile range; [e] SD: Standard deviation

[f] Smoking status describes cigarette smoking; [g] Pathologically confirmed using American Joint Committee on Cancer (AJCC) 7th Edition

[h] Risk stratification based on the 2019 National Comprehensive Cancer Network (NCCN) guideline (see methods)

[i] PSA: Prostate specific antigen

Supplementary Table 2. Lower limit of detection for the 92 immuno-oncological proteins assayed

Serum proteins	Lower Limit of Detection (LLOD) in NPX	Missing Data freq. (proportion of samples with protein level below LLOD)
IL33	1.59195	100%
IL-35	1.18313	100%
IL-21	1.72309	99%
IL2	1.68844	99%
IFN-beta	1.84622	95%
IL13	1.56681	94%
IL-1 alpha	-0.03527	92%
CXCL12	2.22920	87%
IFN-gamma	1.55567	87%
TNF	2.15958	85%
ARG1	5.27490	76%
IL4	0.54505	75%
IL5	2.01127	62%
CD28	2.49576	58%
FGF2	1.24047	43%
NOS3	1.05396	37%
ADGRG1	1.45257	16%
MIC-A/B	1.23883	9%
CD4	-0.13759	5%
IL10	2.30912	5%
CD8A	2.55944	4%
CCL19	2.95390	4%
CXCL10	1.42680	3%
PTN	0.57332	3%
IL12RB1	1.66460	2%
VEGFC	0.05409	2%
MCP-3	1.37635	2%
CXCL5	6.60140	2%
CXCL11	2.22295	1%
PD-L2	1.10516	1%
LAP TGF-beta-1	0.97475	1%
GZMH	2.05211	0%
EGF	4.36494	0%
CRTAM	1.07615	0%
MCP-4	3.14076	0%
CCL4	3.15472	0%
DCN	1.24817	0%
GZMB	1.33242	0%
CD40-L	2.42129	0%
IL7	1.73351	0%
TNFRSF4	2.60002	0%
CASP-8	1.52429	0%
CXCL13	2.32015	0%
IL12	1.44479	0%
ANG-1	3.51630	0%
IL6	2.02073	0%
CXCL9	1.86220	0%
ADA	0.21076	0%
CXCL1	4.26373	0%
MCP-2	2.38761	0%
HO-1	5.73997	0%
CX3CL1	0.63004	0%
CD70	1.10604	0%
CCL23	2.99034	0%
CCL3	2.05152	0%
NCR1	0.66578	0%
CCL17	2.84146	0%
ICOSLG	1.66922	0%
CCL20	2.39470	0%
IL8	2.99315	0%
TNFRSF9	1.21451	0%
TIE2	2.64389	0%
CD244	1.45933	0%
PGF	2.42442	0%
MCP-1	3.78349	0%
TRAIL	1.76455	0%
CAIX	0.77464	0%
Gal-9	2.49023	0%
VEGFR-2	0.95838	0%
CD40	4.25081	0%
IL18	3.18339	0%
TNFSF14	2.16990	0%
TWEAK	2.51722	0%
PDGF subunit B	4.82460	0%
PDCD1	1.80311	0%
FASLG	1.83999	0%
Gal-1	1.76849	0%
PD-L1	2.05346	0%
CD27	2.43845	0%
HGF	2.23412	0%
GZMA	0.65531	0%
TNFRSF12A	1.92242	0%
CD5	1.08175	0%
MMP7	5.36396	0%
TNFRSF21	2.48109	0%
ANGPT2	1.12478	0%
LAMP3	2.20090	0%
MMP12	0.69984	0%
VEGFA	2.15947	0%
KLRD1	1.67210	0%
CD83	1.33464	0%
CSF-1	1.43577	0%

Supplementary Table 3. List of 82 immuno-oncological proteins detected in more than 20% of the serum samples

Protein symbol	Protein name	Uniprot ID
IL8	Interleukin-8	P10145
TNFRSF9	Tumor necrosis factor receptor superfamily member 9	Q07011
TIE2	Angiopoietin-1 receptor	Q02763
MCP-3	Monocyte chemotactic protein 3	P80098
CD40-L	CD40 ligand	P29965
CD244	Natural killer cell receptor 2B4	Q9BZW8
EGF	Pro-epidermal growth factor	P01133
ANG-1	Angiopoietin-1	Q15389
IL7	Interleukin-7	P13232
PGF	Placenta growth factor	P49763
IL6	Interleukin-6	P05231
ADGRG1	Adhesion G-protein coupled receptor G1	Q9Y653
MCP-1	Monocyte chemotactic protein 1	P13500
CRTAM	Cytotoxic and regulatory T-cell molecule (CRTAM)	O95727
CXCL11	C-X-C motif chemokine 11	O14625
MCP-4	Monocyte chemotactic protein 4	Q99616
TRAIL	TNF-related apoptosis-inducing ligand	P50591
FGF2	Fibroblast growth factor 2	P09038
CXCL9	C-X-C motif chemokine 9	Q07325
CD8A	T-cell surface glycoprotein CD8 alpha chain	P01732
CAIX	Carbonic anhydrase IX	Q16790
ADA	Adenosine Deaminase	P00813
CD4	T-cell surface glycoprotein CD4	P01730
NOS3	Nitric oxide synthase, endothelial	P29474
Gal-9	Galectin-9	O00182
VEGFR-2	Vascular endothelial growth factor receptor 2	P35968
CD40	CD40L receptor	P25942
IL18	Interleukin-18	Q14116
GZMH	Granzyme H	P20718
VEGFC	Vascular endothelial growth factor C	P49767
LAP TGF-beta-1	Latency-associated peptide transforming growth factor beta-1	P01137
CXCL1	C-X-C motif chemokine 1	P09341
TNFSF14	Tumor necrosis factor ligand superfamily member 14	O43557
TWEAK	Tumor necrosis factor (Ligand) superfamily, member 12	O43508
PDGF subunit B	Platelet-derived growth factor subunit B	P01127
PDCD1	Programmed cell death protein 1	Q15116
FASLG	Fas antigen ligand	P48023
CD28	T-cell-specific surface glycoprotein CD28	P10747
CCL19	C-C motif chemokine 19	Q99731
MCP-2	Monocyte chemotactic protein 2	P80075
CCL4	C-C motif chemokine 4	P13236

Protein symbol	Protein name	Uniprot ID
Gal-1	Galectin-1	P09382
PD-L1	Programmed cell death 1 ligand 1	Q9NZQ7
CD27	CD27 antigen	P26842
CXCL5	C-X-C motif chemokine 5	P42830
IL5	Interleukin-5	P05113
HGF	Hepatocyte growth factor	P14210
GZMA	Granzyme A	P12544
HO-1	Heme oxygenase 1	P09601
CX3CL1	Fractalkine	P78423
CXCL10	C-X-C motif chemokine 10	P02778
CD70	CD70 antigen	P32970
IL10	Interleukin-10	P22301
TNFRSF12A	Tumor necrosis factor receptor superfamily member 12A	Q9NP84
CCL23	C-C motif chemokine 23	P55773
CD5	T-cell surface glycoprotein CD5	P06127
CCL3	C-C motif chemokine 3	P10147
MMP7	Matrix metalloproteinase-7	P09237
NCR1	Natural cytotoxicity triggering receptor 1	O76036
DCN	Decorin	P07585
TNFRSF21	Tumor necrosis factor receptor superfamily member 21	O75509
TNFRSF4	Tumor necrosis factor receptor superfamily member 4	P43489
MIC-A/B	MHC class I polypeptide-related sequence A/B	Q29983,Q29980
CCL17	C-C motif chemokine 17	Q92583
ANGPT2	Angiopoietin-2	O15123
PTN	Pleiotrophin	P21246
LAMP3	Lysosome-associated membrane glycoprotein 3	Q9UQV4
CASP-8	Caspase-8	Q14790
ICOSLG	ICOS ligand	O75144
MMP12	Matrix metalloproteinase-12	P39900
CXCL13	C-X-C motif chemokine 13	O43927
PD-L2	Programmed cell death 1 ligand 2	Q9BQ51
VEGFA	Vascular endothelial growth factor A	P15692
IL4	Interleukin-4	P05112
IL12RB1	Interleukin-12 receptor subunit beta-1	P42701
CCL20	C-C motif chemokine 20	P78556
KLRD1	Natural killer cells antigen CD94	Q13241
GZMB	Granzyme B	P10144
CD83	CD83 antigen	Q01151
IL12	Interleukin-12	P29459,P29460
CSF-1	Macrophage colony-stimulating factor 1	P09603
ARG1	Arginase-1	P05089-1

Supplementary Table 4. Top 10 Pearson pairwise correlations in men with and without prostate cancer

Ghanaian men without prostate cancer

**Serum protein 1**	**Serum Protein 2**	**Correlation (r)**
PDL2	ICOSLG	0.84
ICOSLG	HO1	-0.79
GZMB	GZMH	0.76
HO1	PDL2	-0.73
KLRD1	CD244	0.71
CCL3	CCL4	0.71
EGF	CD40L	0.71
PDL2	TIE2	0.70
CXCL10	CXCL9	0.70
IL8	CASP8	0.69

AA men without prostate cancer

**Serum protein 1**	**Serum Protein 2**	**Correlation (r)**
IL8	MCP3	0.89
CD83	TNFRSF9	0.85
TNFRSF4	TNFRSF9	0.85
EGF	CD40L	0.83
IL8	CASP8	0.82
CD83	TNFRSF4	0.80
PGF	TNFRSF9	0.80
PGF	TNFRSF21	0.80
PGF	TNFRSF4	0.79
PGF	NCR1	0.79

EA men without prostate cancer

**Serum protein 1**	**Serum Protein 2**	**Correlation (r)**
IL8	MCP3	0.89
CCL3	CCL4	0.82
EGF	CD40L	0.80
IL8	CASP8	0.80
TNFRSF4	TNFRSF9	0.80
GZMB	GZMH	0.78
PGF	CD27	0.78
CD83	TNFRSF9	0.76
CD83	TNFRSF9	0.76
MCP3	CASP8	0.75

Ghanaian men with prostate cancer

**Serum protein 1**	**Serum Protein 2**	**Correlation (r)**
CD8A	HO1	0.86
CXCL10	TWEAK	0.86
TNFRSF9	TNFRSF4	0.85
CCL3	CCL4	0.85
TNFRSF9	PGF	0.85
CXCL10	CXCL11	0.85
CD8A	TIE2	-0.84
IL6	CCL4	0.84
TNFRSF4	PGF	0.83
TNFRSF12A	PGF	0.82

AA men with prostate cancer

**Serum protein 1**	**Serum Protein 2**	**Correlation (r)**
MCP3	IL8	0.89
EGF	CD40L	0.87
TNFRSF4	TNFRSF9	0.86
TNFRSF9	PGF	0.84
CD83	TNFRSF9	0.83
CD83	PGF	0.82
PGF	TNFRSF12A	0.82
CD83	TNFRSF4	0.82
TNFRSF4	PGF	0.80
CX3CL1	PGF	0.80

EA men with prostate cancer

**Serum protein 1**	**Serum Protein 2**	**Correlation (r)**
MCP3	IL8	0.84
TNFRSF4	TNFRSF9	0.80
EGF	CD40L	0.77
CCL3	CCL4	0.76
PGF	TNFRSF12A	0.75
TNFRSF21	PGF	0.75
TNFSF14	EGF	0.75
PGF	TNFRSF9	0.74
PGF	TNFRSF4	0.74
MCP3	CASP8	0.75

Supplementary Table 5. The association of blood CRP with clinical/sociodemographic variables estimated using multiple linear regression

	Coefficient [95% CI]	*P*-value	*q*-value
BMI (kg/m2)	59.56 [-29.6, 148.7]	0.19	0.57
age	9.75 [-58.53, 78.02]	0.78	0.96
education (high school or less, some college, college, professional school)	10.9 [-448.8, 427.0]	0.96	0.96
smoke (never, former, vs. current)	891.9 [220.6, 1563.3]	0.01	0.06
diabetes (no vs. yes)	-95.5 [-1391.8, 1200.8]	0.88	0.96
PSA (ng/ml)	-59.3[-507.5, 389.0]	0.79	0.96

Supplementary Table 6. The fraction of variance in each of the serum proteins explained by degree of West African ancestry

Serum Proteins	Number of observations	Root Mean Square Error (RMSE)	Variance fraction	F-statistics	*p* -value	†*q-value*
CXCL5	795	0.699	0.498	786.48	**<0.0001**	**<0.0001**
CXCL1	795	0.632	0.408	545.60	**<0.0001**	**<0.0001**
MCP2	795	0.648	0.152	141.88	**<0.0001**	**<0.0001**
MCP1	795	0.666	0.147	136.34	**<0.0001**	**<0.0001**
CXCL11	795	1.071	0.128	116.81	**<0.0001**	**<0.0001**
CCL23	795	0.521	0.105	92.68	**<0.0001**	**<0.0001**
PTN	795	1.153	0.100	87.66	**<0.0001**	**<0.0001**
TWEAK	795	0.332	0.090	78.73	**<0.0001**	**<0.0001**
NCR1	795	0.556	0.074	63.32	**<0.0001**	**<0.0001**
CCL17	795	0.781	0.049	40.57	**<0.0001**	**<0.0001**
IL18	795	0.610	0.048	40.11	**<0.0001**	**<0.0001**
CD8A	795	0.790	0.047	38.81	**<0.0001**	**<0.0001**
IL12	795	0.792	0.042	34.53	**<0.0001**	**<0.0001**
CXCL9	795	0.910	0.041	34.04	**<0.0001**	**<0.0001**
TNFRSF4	795	0.509	0.041	33.50	**<0.0001**	**<0.0001**
MCP4	795	0.697	0.039	32.23	**<0.0001**	**<0.0001**
MMP7	795	0.308	0.030	24.28	**<0.0001**	**<0.0001**
CCL3	795	1.464	0.029	23.38	**<0.0001**	**<0.0001**
TNFRSF21	795	0.270	0.024	19.66	**<0.0001**	**<0.0001**
TNFRSF9	795	0.561	0.024	19.44	**<0.0001**	**<0.0001**
HO1	795	0.395	0.023	18.96	**<0.0001**	**0.0001**
PDL2	795	0.405	0.022	17.61	**<0.0001**	**0.0001**
CD70	795	0.460	0.020	16.13	**0.0001**	**0.0002**
CCL20	795	1.255	0.020	16.02	**0.0001**	**0.0002**
TNFSF14	795	0.904	0.019	15.07	**0.0001**	**0.0004**
IL7	795	0.592	0.018	14.74	**0.0001**	**0.0004**
ARG1	795	0.372	0.017	14.04	**0.0002**	**0.0006**
CCL19	795	0.892	0.016	13.15	**0.0003**	**0.0009**
LAMP3	795	0.753	0.012	9.22	**0.0025**	**0.0070**
TRAIL	795	0.326	0.011	8.96	**0.0028**	**0.0078**
HGF	795	0.480	0.011	8.62	**0.0034**	**0.0091**
FGF2	795	0.624	0.010	8.31	**0.004**	**0.0104**
CASP8	795	1.250	0.009	7.51	**0.0063**	**0.0156**
GZMB	795	0.966	0.008	6.59	**0.0104**	**0.0251**
CCL4	795	0.835	0.008	6.53	**0.0108**	**0.0253**
ICOSLG	795	0.286	0.008	6.19	**0.0131**	**0.0290**
PGF	795	0.415	0.008	6.19	**0.0131**	**0.0290**
NOS3	795	0.381	0.007	5.60	**0.0182**	**0.0393**
MICAB	795	1.682	0.007	5.52	**0.0191**	**0.0401**
GZMH	795	1.014	0.006	4.56	**0.033**	0.0677
PDCD1	795	0.637	0.006	4.52	**0.0339**	0.0677
CD83	795	0.432	0.005	4.30	**0.0384**	0.0732
FASLG	795	0.437	0.005	4.32	**0.038**	0.0732
CRTAM	795	0.599	0.005	4.03	**0.0451**	0.0822
LAPTGFbeta1	795	0.336	0.005	4.06	**0.0441**	0.0822
CSF1	795	0.239	0.005	3.82	0.0509	0.0907
CXCL10	795	0.905	0.005	3.72	0.0542	0.0946
Gal9	795	0.402	0.004	3.41	0.0653	0.1116
VEGFC	795	0.469	0.004	3.29	0.0702	0.1174
PDGFsubunitB	795	0.127	0.004	3.12	0.0778	0.1275
IL6	795	1.181	0.004	2.91	0.0885	0.1423
Gal1	795	0.238	0.004	2.87	0.0905	0.1426
ANG1	795	0.292	0.003	2.21	0.1377	0.2130
IL10	795	0.550	0.002	1.80	0.1807	0.2694
KLRD1	795	0.615	0.002	1.81	0.1789	0.2694
CD27	795	0.466	0.002	1.75	0.1859	0.2722
CX3CL1	795	0.446	0.002	1.56	0.2128	0.3061
IL8	795	2.693	0.002	1.46	0.227	0.3210
VEGFR2	795	0.259	0.002	1.19	0.2754	0.3828
CD5	795	0.410	0.001	1.14	0.2858	0.3906
CD40	795	0.477	0.001	1.00	0.3187	0.4284
CD28	795	0.355	0.001	0.94	0.3323	0.4395
TNFRSF12A	795	0.479	0.001	0.77	0.3792	0.4935
IL4	795	0.938	0.001	0.72	0.3949	0.5059
ADA	795	0.594	0.001	0.61	0.4354	0.5275
ANGPT2	795	0.500	0.001	0.60	0.4385	0.5275
CD40L	795	0.844	0.001	0.63	0.4283	0.5275
MCP3	795	2.432	0.001	0.61	0.4359	0.5275
TIE2	795	0.267	0.001	0.59	0.4439	0.5275
EGF	795	1.008	0.001	0.55	0.4581	0.5367
CD4	795	0.372	0.001	0.38	0.5376	0.6111
IL5	795	1.320	0.001	0.37	0.5441	0.6111
VEGFA	795	0.515	0.001	0.37	0.5408	0.6111
GZMA	795	0.484	0.000	0.17	0.6839	0.7578
CAIX	795	0.651	0.000	0.13	0.7201	0.7873
MMP12	795	0.763	0.000	0.08	0.7808	0.8424
ADGRG1	795	1.053	0.000	0.05	0.8243	0.8779
CD244	795	0.376	0.000	0.03	0.8551	0.8990
PDL1	795	0.551	0.000	0.02	0.8862	0.9199
CXCL13	795	0.619	0.000	0.00	0.9678	0.9879
DCN	795	0.278	0.000	0.00	0.9841	0.9879
IL12RB1	795	0.433	0.000	0.00	0.9879	0.9879

Analysis of variance was performed for the levels of each of the 82 immune-oncological cytokines as a function of genetic estimation of West African admixture among men without prostate cancer from the NCI-Maryland study.

†The q-values represent FDR-adjusted p-values for multiple-testing of n=82.

Supplementary Table 7. The fraction of variance in each of the serum proteins explained by degree of West African ancestry after adjusting for differences in age, BMI, aspirin use, education, diabetes status, smoking, and income

Serum Proteins	Number of observations	Root Mean Square Error (RMSE)	Variance fraction	F-statistics	p-value	q-value
CXCL5	726	0.703	0.501	725.43	**<0.0001**	**<0.0001**
CXCL1	726	0.628	0.406	494.33	**<0.0001**	**<0.0001**
MCP1	726	0.653	0.165	142.58	**<0.0001**	**<0.0001**
MCP2	726	0.649	0.146	123.35	**<0.0001**	**<0.0001**
CXCL11	726	1.056	0.130	108.20	**<0.0001**	**<0.0001**
PTN	726	1.142	0.111	90.36	**<0.0001**	**<0.0001**
CCL23	726	0.522	0.108	87.17	**<0.0001**	**<0.0001**
TWEAK	726	0.331	0.087	68.55	**<0.0001**	**<0.0001**
NCR1	726	0.556	0.076	59.19	**<0.0001**	**<0.0001**
IL18	726	0.602	0.056	43.19	**<0.0001**	**<0.0001**
CCL17	726	0.778	0.054	40.90	**<0.0001**	**<0.0001**
CD8A	726	0.789	0.050	38.29	**<0.0001**	**<0.0001**
IL12	726	0.776	0.048	36.62	**<0.0001**	**<0.0001**
TNFRSF4	726	0.509	0.048	36.41	**<0.0001**	**<0.0001**
CXCL9	726	0.886	0.048	36.37	**<0.0001**	**<0.0001**
MCP4	726	0.700	0.037	27.65	**<0.0001**	**<0.0001**
MMP7	726	0.307	0.028	20.82	**<0.0001**	**<0.0001**
TNFRSF9	726	0.562	0.026	19.31	**<0.0001**	**0.0001**
HO1	726	0.396	0.025	18.69	**<0.0001**	**0.0001**
TNFSF14	726	0.908	0.024	17.76	**<0.0001**	**0.0001**
TNFRSF21	726	0.271	0.024	17.64	**<0.0001**	**0.0001**
CCL3	726	1.432	0.022	16.07	**0.0001**	**0.0002**
CD70	726	0.457	0.022	16.01	**0.0001**	**0.0002**
PDL2	726	0.408	0.021	15.25	**0.0001**	**0.0004**
ARG1	726	0.375	0.019	13.83	**0.0002**	**0.0007**
HGF	726	0.481	0.016	11.69	**0.0007**	**0.0021**
TRAIL	726	0.325	0.016	11.63	**0.0007**	**0.0021**
IL7	726	0.592	0.015	11.11	**0.0009**	**0.0026**
CCL19	726	0.892	0.014	9.89	**0.0017**	**0.0049**
CCL20	726	1.193	0.013	9.80	**0.0018**	**0.0050**
CASP8	726	1.244	0.013	9.58	**0.002**	**0.0052**
LAMP3	726	0.753	0.013	9.59	**0.002**	**0.0052**
FGF2	726	0.630	0.011	8.12	**0.0045**	**0.0112**
ICOSLG	726	0.287	0.011	7.96	**0.0049**	**0.0118**
GZMB	726	0.967	0.009	6.53	**0.0108**	**0.0254**
CXCL10	726	0.887	0.008	5.88	**0.0156**	**0.0355**
PGF	726	0.417	0.008	5.59	**0.0183**	**0.0405**
NOS3	726	0.372	0.006	4.59	**0.0325**	0.0701
CD83	726	0.435	0.006	4.36	**0.0372**	0.0783
LAPTGFbeta1	726	0.339	0.006	4.31	**0.0382**	0.0783
GZMH	726	1.021	0.006	4.02	**0.0454**	0.0887
PDCD1	726	0.646	0.006	4.03	**0.0451**	0.0887
MICAB	726	1.661	0.005	3.68	0.0556	0.1039
Gal9	726	0.401	0.005	3.67	0.0558	0.1039
CSF1	726	0.240	0.005	3.56	0.0595	0.1085
CRTAM	726	0.604	0.005	3.39	0.0661	0.1179
VEGFC	726	0.469	0.004	3.07	0.0804	0.1402
CCL4	726	0.812	0.004	2.94	0.0869	0.1484
FASLG	726	0.439	0.004	2.90	0.0889	0.1488
VEGFR2	726	0.257	0.003	2.36	0.1248	0.2047
IL8	726	2.665	0.003	2.27	0.1325	0.2131
Gal1	726	0.240	0.003	2.19	0.1394	0.2199
CD40	726	0.481	0.002	1.55	0.2135	0.3303
IL5	726	1.278	0.002	1.36	0.2436	0.3688
CD5	726	0.409	0.002	1.30	0.2546	0.3728
IL10	726	0.541	0.002	1.34	0.2473	0.3688
CD28	726	0.367	0.002	1.25	0.2643	0.3745
PDGFsubunitB	726	0.127	0.002	1.24	0.2649	0.3745
ANG1	726	0.296	0.002	1.10	0.2949	0.4054
CD27	726	0.464	0.002	1.09	0.2966	0.4054
MCP3	726	2.417	0.002	1.06	0.3044	0.4092
ADA	726	0.576	0.001	0.92	0.3386	0.4407
CX3CL1	726	0.442	0.001	0.92	0.3381	0.4407
IL4	726	0.965	0.001	0.87	0.3503	0.4489
KLRD1	726	0.613	0.001	0.83	0.3616	0.4562
CD40L	726	0.861	0.001	0.75	0.3873	0.4812
IL6	726	1.144	0.001	0.69	0.4053	0.4961
TIE2	726	0.265	0.001	0.55	0.4585	0.5528
EGF	726	1.029	0.001	0.33	0.564	0.6607
IL12RB1	726	0.432	0.001	0.34	0.5576	0.6607
CAIX	726	0.647	0.000	0.30	0.5864	0.6624
CD4	726	0.369	0.000	0.31	0.5773	0.6624
VEGFA	726	0.521	0.000	0.29	0.5897	0.6624
DCN	726	0.278	0.000	0.13	0.7209	0.7882
TNFRSF12A	726	0.483	0.000	0.15	0.701	0.7768
CXCL13	726	0.609	0.000	0.09	0.7663	0.8268
GZMA	726	0.481	0.000	0.04	0.8445	0.8993
ADGRG1	726	1.042	0.000	0.00	0.9783	0.9783
ANGPT2	726	0.497	0.000	0.02	0.8948	0.9058
CD244	726	0.377	0.000	0.02	0.8921	0.9058
MMP12	726	0.757	0.000	0.02	0.8818	0.9058
PDL1	726	0.562	0.000	0.02	0.8764	0.9058

Analysis of variance was performed for the levels of each of the 82 immune-oncological cytokines as a function of genetic estimation of West African admixture among men without prostate cancer from the NCI-Maryland study.
†The q-values represent FDR-adjusted p-values for multiple-testing of n=82.

Supplementary Table 8. Immune oncological markers that are significantly elevated in both Ghanaian and AA men compared to EA men

markers	EA median (IQR) (linear NPX)	AA median (IQR) (linear NPX)	Ghanaian median (IQR) (linear NPX)	median ratio (AA/EA)	p-value	q-value	median ratio (Ghanaian/EA)	p-value	q-value
CXCL5	6284.05 (4332.56-8943.36)	17648.06 (13969.29-19884.74)	18950.86 (16678.05-21055.39)	2.81	<0.0001	<0.0001	3.02	<0.0001	<0.0001
CXCL1	1794.85 (1347.21-2317.20)	3838.60 (2781.05-5121.68)	4730.16 (3864.12-5615.48)	2.14	<0.0001	<0.0001	2.64	<0.0001	<0.0001
CXCL11	199.81 (140.72-287.12)	390.17 (187.00-674.34)	686.85 (400.24-1293.64)	1.95	<0.0001	<0.0001	3.44	<0.0001	<0.0001
MCP2	401.48 (306.12-535.24)	570.79 (447.25-721.71)	609.02 (479.80-788.77)	1.42	<0.0001	<0.0001	1.52	<0.0001	<0.0001
CCL17	1144.06 (806.42-1613.84)	1515.00 (1077.18-2151.31)	1578.03 (1177.14-2094.19)	1.32	<0.0001	<0.0001	1.38	<0.0001	<0.0001
MCP4	1076.23 (792.86-1444.83)	1358.71 (931.83-1825.48)	1384.04 (1025.71-1929.18)	1.26	<0.0001	<0.0001	1.29	<0.0001	<0.0001
CD70	21.69 (17.71-27.17)	24.56 (20.03-29.46)	32.51 (27.01-38.49)	1.13	<0.0001	0.0001	1.5	<0.0001	<0.0001
PDL2	10.34 (8.69-12.02)	11.50 (9.41-13.74)	15.47 (12.02-28.00)	1.11	0.0120	0.0304	1.5	<0.0001	<0.0001
MMP7	3836.16 (3309.16-4334.54)	4204.66 (3677.46-4856.59)	4470.14 (3889.38-5087.45)	1.10	<0.0001	<0.0001	1.17	<0.0001	<0.0001
CCL19	1075.95 (855.70-1458.43)	1177.98 (854.58-1946.63)	1170.39 (825.52-1615.30)	1.09	0.0194	0.0449	1.09	0.0352	0.0419

Medians were compared using two-sided Wilcoxon rank sum tests.

†The q-values represent FDR-adjusted p-values for multiple-testing.

Supplementary Table 9. Immune oncological markers that are significantly downregulated in both Ghanaian and AA men compared to EA men

markers	EA median (IQR)	AA median (IQR)	Ghanaian median (IQR)	median ratio (AA/EA)	p-value	†q-value	median ratio (Ghanaian/EA)	p-value	†q-value
MCP1	5013.63 (4088.21-6276.75)	3168.62 (2454.90-5211.40)	2316.18 (1999.64-2779.57)	0.63	<0.0001	<0.0001	0.46	<0.0001	<0.0001
IL12	189.78 (136.00-251.02)	134.41 (100.24-187.47)	146.88 (112.96-198.63)	0.71	<0.0001	<0.0001	0.77	<0.0001	<0.0001
CCL23	2303.56 (1869.12-2871.36)	1735.11 (1397.11-2407.05)	1803.47 (1408.06-2344.48)	0.75	<0.0001	<0.0001	0.78	<0.0001	<0.0001
CD8A	1172.34 (859.33-1746.96)	886.06 (674.28-1337.39)	938.37 (586.78-1367.73)	0.76	<0.0001	<0.0001	0.80	<0.0001	<0.0001
NCR1	14.57 (12.02-18.01)	11.63 (9.37-14.23)	10.12 (8.27-12.53)	0.80	<0.0001	<0.0001	0.69	<0.0001	<0.0001
TNFRSF4	26.63 (22.81-31.37)	21.85 (18.42-27.95)	21.90 (17.85-27.63)	0.82	<0.0001	<0.0001	0.82	<0.0001	<0.0001
TNFSF14	140.12 (91.45-210.43)	117.88 (76.88-185.51)	116.26 (84.07-164.84)	0.84	0.0003	0.0010397	0.83	0.0001	0.0002
TWEAK	1031.16 (868.16-1186.18)	872.11 (762.15-1009.09)	889.01 (761.36-1018.81)	0.85	<0.0001	<0.0001	0.86	<0.0001	<0.0001
IL7	96.82 (74.47-127.65)	85.91 (66.26-109.86)	84.68 (65.48-103.35)	0.89	<0.0001	0.0002	0.87	<0.0001	<0.0001
HGF	684.74 (566.67-840.30)	632.71 (533.60-775.66)	612.32 (513.02-734.06)	0.92	0.00162	0.0050	0.89	<0.0001	<0.0001
HO1	7742.15 (6288.28-8830.06)	7200.42 (5827.13-8554.93)	4844.86 (2283.44-6817.24)	0.93	0.0164	0.0390	0.63	<0.0001	<0.0001
TNFRSF21	548.59 (497.35-598.66)	511.27 (456.66-583.68)	489.92 (435.65-557.77)	0.93	<0.0001	<0.0001	0.89	<0.0001	<0.0001
ANG1	2480.61 (2221.52-2734.09)	2397.11 (2139.86-2647.99)	2351.09 (2033.01-2626.74)	0.97	0.0154	0.0378	0.95	0.0003	0.0004

Medians were compared using two-sided Wilcoxon rank sum tests.

†The q-values represent FDR-adjusted p-values for multiple-testing.

Supplementary Table 10. Serum proteins grouped by biological process

Apoptosis	Autophagy	Chemotaxis	Promote TI	Suppress TI	Vasculature
Gal9	ADA	CCL17	CX3CL1	CXCL11	CXCL11
TNFRSF12A	CAIX	CCL19	CXCL10	CXCL13	CXCL1
TWEAK	HO1	CCL20	CXCL11	CD4	CXCL5
MMP7		CCL23	CXCL13	CD5	IL8
CD40L		CCL3	CD40L	IL18	MCP4
TRAIL		CCL4	TRAIL	IL6	Gal9
CASP8		CX3CL1	CD244	CCL17	Gal1
FASLG		CXCL1	CD27	CCL19	MMP12
GZMA		CXCL10	CD28	CCL20	CXCL10
GZMB		CXCL11	CD4	CXCL1	CXCL9
GZMH		CXCL13	CD40	CXCL5	CCL23
TNFRSF21		CXCL5	CD5	IL8	MCP1
		IL8	CD70	MCP4	CAIX
		MCP1	CD83	Gal9	TNFRSF12A
		MCP2	CD8A	MMP7	TWEAK
		MCP3	CRTAM	CSF1	ADGRG1
		MCP4	CXCL9	Gal1	ANG1
			ICOSLG	IL10	ANGPT2
			IL12RB1	IL4	DCN
			IL18	IL5	EGF
			IL6	LAMP3	FGF2
			IL7	LAPTGFbeta1	HGF
			KLRD1	MICAB	IL12
			NCR1	MMP12	NOS3
			TNFRSF4	PDCD1	PDGFsubunitB
			TNFRSF9	PDL1	PGF
			TNFSF14	PDL2	PTN
				ARG1	TIE2
					VEGFA
					VEGFC
					VEGFR2

Supplementary Table 11. Effect of biological processes scores on all-cause mortality of prostate cancer patients

Biological Processes score	Unadjusted Hazard Ratio (95%CI)	p-value	q-value	*Adjusted Hazard Ratio (95%CI)	*Adjusted Hazard Ratio (99%CI)	p-value	†q-value
**Apoptosis score**	0.75 (0.35, 1.59)	0.449	0.659	0.94 (0.43, 2.06)	0.94 (0.34, 2.64)	0.881	0.969
**Autophagy score**	1.12 (0.78, 1.61)	0.549	0.659	0.92 (0.62, 1.34)	0.92 (0.55, 1.52)	0.651	0.969
**Chemotaxis score**	0.57 (0.25, 1.30)	0.180	0.539	0.46 (0.18, 1.17)	0.46 (0.13, 1.57)	0.102	0.306
**Promote Tumor Immunity Score**	0.93 (0.49, 1.78)	0.829	0.829	0.95 (0.46, 1.94)	0.95 (0.37, 2.44)	0.883	0.969
**Suppress Tumor Immunity Score**	**5.66 (2.07, 15.48)**	**0.001**	**0.004**	**5.96 (1.87, 18.95)**	**5.96 (1.30, 27.26)**	**0.002**	**0.012**
**Vasculature score**	1.46 (0.58, 3.69)	0.421	0.659	1.02 (0.38, 2.75)	1.02 (0.28, 3.76)	0.969	0.969

Two-sided multivariable Cox regression analyses were used to assess if the pathways were independently associated with survival.

*Hazard ratios were adjusted for age, BMI, race/ethnicity, education level, income level, smoking status, diabetes status, aspirin use, treatment type, and NCCN risk score.

†The q-values represent FDR-adjusted p-values for multiple testing of n=6.

Supplementary Table 12. Effect of biological processes scores on all-cause mortality of population controls

Biological Processes scores	Unadjusted Hazard Ratio (95%CI)	p-value	q-value	*Adjusted Hazard Ratio (95%CI)	*Adjusted Hazard Ratio (99%CI)	p-value	†q-value
**Apoptosis score**	**0.27 (0.10, 0.75)**	**0.012**	0.059	0.47 (0.16, 1.33)	0.47 (0.12, 1.84)	0.153	0.458
**Autophagy score**	1.19 (0.76, 1.87)	0.457	0.457	1.25 (0.79, 2.00)	1.25 (0.68, 2.31)	0.344	0.516
**Chemotaxis score**	0.40 (0.15, 1.11)	0.078	0.117	0.77 (0.24, 2.42)	0.77 (0.17, 3.48)	0.649	0.779
**Promote Tumor Immunity Score**	**2.84 (1.18, 6.81)**	**0.020**	0.059	2.04 (0.82, 5.07)	2.04 (0.61, 6.75)	0.127	0.458
**Suppress Tumor Immunity Score**	3.60 (0.90, 14.44)	0.071	0.117	2.19 (0.48, 9.96)	2.19 (0.30, 16.03)	0.310	0.516
**Vasculature score**	2.74 (0.70, 10.76)	0.148	0.178	1.12 (0.25, 5.10)	1.12 (0.15, 8.21)	0.866	0.886

Two-sided multivariable Cox regression analyses were used to assess if the pathways were independently associated with survival.
*The hazard ratio was adjusted for age, BMI, education level, smoking status, diabetes status, aspirin use, income level, and race/ethnicity.
†The q-values represent FDR-adjusted p-values for multiple testing of n=6.

Supplementary Table 13. Effect of biological processes scores on prostate cancer-specific mortality

Biological Processes scores	Unadjusted Hazard Ratio (95% CI)	p-value	q-value	*Adjusted Hazard Ratio (95% CI)	*Adjusted Hazard Ratio (99% CI)	p-value	†q-value
**Apoptosis score**	0.56 (0.13, 2.30)	0.417	0.787	0.92 (0.23, 3.73)	0.92 (0.15, 5.80)	0.907	0.907
**Autophagy score**	1.20 (0.60, 2.39)	0.601	0.787	1.08 (0.52, 2.24)	1.08(0.41, 2.81)	0.837	0.907
**Chemotaxis score**	1.38 (0.30, 6.42)	0.683	0.787	0.47 (0.08, 2.74)	0.47 (0.05, 4.77)	0.398	0.907
**Promote Tumor Immunity Score**	1.32 (0.39, 4.49)	0.652	0.787	0.70 (0.18, 2.69)	0.70 (0.12, 4.11)	0.607	0.907
**Suppress Tumor Immunity Score**	3.69 (0.55, 24.82)	0.179	0.787	**11.94 (1.36, 105.21)**	11.94 (0.68, 208.46)	**0.026**	0.156
**Vasculature score**	0.79 (0.14, 4.41)	0.787	0.787	0.50 (0.07, 3.70)	0.50 (0.04, 6.96)	0.493	0.907

Two-sided multivariable Cox regression analyses were used to assess if the pathways were independently associated with survival.
*Hazard ratios were adjusted for age, BMI, race/ethnicity, education level, income level, smoking status, diabetes status, aspirin use, treatment type, and NCCN risk score.

†The q-values represent FDR-adjusted p-values for multiple testing of n=6.

Supplementary Table 14. Effect of biological processes scores on mortality from any cancer following a diagnosis with prostate cancer

Biological Processes score	Unadjusted Hazard Ratio (95% CI)	p-value	q-value	*Adjusted Hazard Ratio (95% CI)	*Adjusted Hazard Ratio (99% CI)	p-value	†q-value
**Apoptosis score**	0.61 (0.21, 1.78)	0.365	0.849	0.83 (0.28, 2.47)	0.83 (0.20, 3.48)	0.733	0.957
**Autophagy score**	1.22 (0.73, 2.04)	0.437	0.849	1.01 (0.60, 1.72)	1.01(0.51, 2.03)	0.957	0.957
**Chemotaxis score**	0.73 (0.23, 2.34)	0.598	0.849	0.34 (0.09, 1.28)	0.34 (0.06, 1.94)	0.111	0.333
**Promote Tumor Immunity Score**	0.83 (0.32, 2.15)	0.708	0.849	0.66 (0.24, 1.85)	0.66 (0.17, 2.56)	0.428	0.856
**Suppress Tumor Immunity Score**	**5.67 (1.38, 23.21)**	**0.016**	0.095	**11.86 (2.35, 59.93)**	**11.86 (1.41, 99.69)**	**0.003**	**0.018**
**Vasculature score**	1.14 (0.31, 4.20)	0.849	0.849	0.87 (0.21, 3.67)	0.87 (0.13, 5.77)	0.852	0.957

Two-sided multivariable Cox regression analyses were used to assess if the pathways were independently associated with survival.
*Hazard ratios were adjusted for age, BMI, race/ethnicity, education level, income level, smoking status, diabetes status, aspirin use, treatment type, and NCCN risk score.
†The q-values represent FDR-adjusted p-values for multiple-testing of n=6.
