# Supplementary material for: Serum proteomics links suppression of tumor immunity to ancestry and lethal prostate cancer
Source: Nat Commun. 2022 Apr 1;13:1759. doi: 10.1038/s41467-022-29235-2 (PMC8975871; doi:10.1038/s41467-022-29235-2)
Supplement: Supplementary file 4 — Description of Additional Supplementary Files [file 41467_2022_29235_MOESM4_ESM.docx]

**Title:** Supplementary Data 1.

**Description:** Association of clinical/socio-demographic characteristics with immune-oncological

proteins

**Title:** Supplementary Data 2.

**Description:** Regression coefficients with confidence intervals for the fraction of variance in

each of the serum proteins explained by degree of West African ancestry

**Title:** Supplementary Data 3.

**Description:** Regression coefficients with confidence intervals for the fraction of variance in

each of the serum proteins explained by degree of West African ancestry after adjusting for

differences in age, BMI, aspirin use, education, diabetes status, smoking, and income

**Title:** Supplementary Data 4.

**Description:** Elastic net Cox regression results for the African American cohort using 82

immune-oncology markers and 6 patient feature covariates (age, education, BMI, smoking

history, aspirin use, and diabetes)

**Title:** Supplementary Data 5.

**Description:** Elastic net Cox regression results for the African American cohort using 82

immune-oncology markers and 6 patient feature covariates (age, education, BMI, smoking

history, aspirin use, diabetes, and NCCN risk score)

**Title:** Supplementary Data 6.

**Description:** West African ancestry estimates obtained with either 100 ancestry informative markers (AIM) or with GWAS-based SNPs (n = 55446)
